# miR-145-5p Inhibits the Proliferation, Migration, and Invasion of Esophageal Carcinoma Cells by Targeting ABRACL

**DOI:** 10.1155/2021/6692544

**Published:** 2021-02-26

**Authors:** Shengming Fan, Pei Chen, Shugang Li

**Affiliations:** Department of Thoracic Surgery, People's Hospital of Anji, 313300 Huzhou, China

## Abstract

**Objective:**

The study is aimed at investigating the regulatory relationship between miR-145-5p and ABRACL, and has tried at clarifying the mechanisms underlying the proliferation, migration, and invasion of esophageal carcinoma (EC) cells.

**Methods:**

Gene expression data related to EC were accessed from TCGA database, and the “edgeR” package was used to screen differentially expressed genes. TargetScan, miRDB, and miRTarBase databases were used to predict potential targets for the target miRNA miR-145-5p. qRT-PCR and Western blot were performed to assess the expression of miR-145-5p and ABRACL in EC cells. Dual-luciferase reporter assay was performed to validate the targeting relationship between miR-145-5p and ABRACL. Functional experiments including CCK-8 assay, Transwell migration, and invasion assays were used to detect the proliferation, migration, and invasion of EC cells.

**Results:**

The expression of miR-145-5p was significantly decreased in EC, while ABRACL was remarkably increased. In addition, there was a negative correlation identified between miR-145-5p and ABRACL mRNA. Overexpressing miR-145-5p was able to suppress cell proliferation, migration, and invasion, whereas silencing miR-145-5p posed an opposite effect. In the meantime, ABRACL was identified as a direct target of miR-145-5p by dual-luciferase reporter assay. Furthermore, miR-145-5p could inhibit the expression of ABRACL, in turn inhibiting the proliferation, migration, and invasion of EC cells.

**Conclusion:**

miR-145-5p functions on the proliferation, migration, and invasion of EC cells via targeting ABRACL, and it may be a novel therapeutic target in EC treatment.

## 1. Introduction

Esophageal carcinoma (EC) is a common gastrointestinal tumor characterized by a high incidence around the world, and it is the sixth most common cause leading to cancer-related death [1]. Lung cancer, gastric cancer, hepatocellular carcinoma, and EC are the four most common cancers in China. Among them, EC is the most prevalent disease and its incidence gradually increases [2]. As there are no typical clinical symptoms manifested in early stages of EC, the illness of many patients may progress to an advanced stage when they are initially diagnosed, and that is the main reason for a high mortality. Special local vascular structure and abundant lymphatic capillaries are favorable factors for migration of EC cells, which contributes to the fact that most people are confirmed with EC accompanied by metastasis. Esophageal squamous cell carcinoma (ESCC) is the most common type of EC with a relatively high incidence in China [3, 4]. At present, treatment of EC is mainly based on surgery combined with radiotherapy or chemotherapy. Such treatment can have a significant effect on the progress of the illness, yet the overall prognosis is still poor. Therefore, identifying effective therapeutic targets is of great significance for the treatment of EC.

MicroRNAs (miRNAs) are small noncoding RNA molecules that regulate translation or degradation of mRNA by binding to the 3′-UTR of their target mRNAs [5, 6]. miRNAs play pivotal roles in tumors including EC by acting as oncogenes or tumor suppressor genes. For example, miR-124-3p directly targets the 3′-UTR region of BCAT1 in ESCC, and downregulation of miR-124-3p is highly correlated with ESCC cell proliferation and migration [7]. In addition, miR-125b was reported to be able to negatively regulate the expression of BCL-2-modifying factor (BMF) in ESCC by interacting with the 3′-UTR within BMF, and overexpressing miR-125b can significantly inhibit the growth of ESCC and induce apoptosis [8]. miR-145-5p, a crucial member of the miRNA family, was identified to be involved in malignant progression of a variety of cancers, such as colorectal cancer [9], breast cancer [10], and gastric cancer [11], and has fantastic potential applications in the field of cancer.

The human ABRACL (ABPA C-terminal like) gene locates on chromosome 6 and encodes a small protein of 81 amino acids, which can enhance actin activity and cell viability [12]. As an atypical winged-helix protein previously named as HSPC280, ABRACL belongs to a new family of low-molecular-weight proteins and is only present in eukaryotes but absent in fungi, with highly conserved sequences across different species [13]. A mouse study conducted by Stylianopoulou et al. found that ABRACL is a nucleoprotein that inhibits neuronal differentiation *in vitro* when it is overexpressed in Neuro2a cells, suggesting its involvement in the regulation of neural progenitor cell proliferation [14]. A report showed that ABRACL can be detected in uterine aspirate of endometrial cancer rather than in that of healthy uterus [15]. Besides, ABRACL was proven to have elevated expression in gastric cancer tissue relative to that in normal tissue, while increased ABRACL indicates unfavorable clinical outcomes of cancer sufferers [16]. However, the role of ABRACL in EC has not been reported.

The aim of this study was to investigate the expression of miR-145-5p in EC cells and its role in cell proliferation, migration, and invasion, and also tried to clarify the underlying mechanisms.

## 2. Materials and Methods

### 2.1. Bioinformatics Analysis

Gene and miRNA Expression Quantification Data related to EC were accessed from TCGA-ESCA (esophageal carcinoma) dataset in TCGA (https://portal.gdc.cancer.gov/). Package “edgeR” was used to identify differentially expressed genes (DEGs), with the threshold set as ∣logFC | >1 and padj < 0.05. TargetScan (http://www.targetscan.org/vert_72/), miRTarBase (http://mirtarbase.mbc.nctu.edu.tw/php/index.php), and miRDB (http://www.mirdb.org/) databases were used to predict downstream target mRNAs for the target miRNA, and a Venn diagram was plotted to find the target mRNA. Survival significance of the target mRNA was evaluated using the clinical information obtained from TCGA.

### 2.2. Cell Culture

Normal human esophageal epithelial cell line HET-1A was purchased from Riken BioResource Center (Tsukuba, Japan). Five EC cell lines Eca-109, EC9706, KYSE150, KYSE180, and BIC-1 were purchased from Cell Center of the Shanghai Institutes for Biological Sciences, Chinese Academy of Sciences (Shanghai, China). All cells were cultured in RPMI-1640 medium (Gibco, USA) containing 10% fetal bovine serum (FBS; Gibco), streptomycin (100 mg/ml; Gibco), and penicillin (100 units/ml; Gibco), and maintained in an incubator at 37°C with 5% CO_2_.

### 2.3. Vector Construction and Cell Transfection

MiR-145-5p mimic, miR-145-5p inhibitor, corresponding negative control and si-abracl respectively purchased from GenePharma (Shanghai, China) were transiently transfected into EC cells by Lipofectamine 2000 (Thermo Fisher Scientific, Inc.), and then maintained in corresponding medium under the environment of 5% CO_2_ at 37°C. The lentiviral expression vector pLVX-IRES-neo (Clontech) was used to establish ABRACL-overexpressed vector oe-ABRACL, which was then used to infect cancer cells, with the virus particle as the negative control. Sequences of the materials for transfection are as follows: miR-145-5p mimic sense: 5′-GUCCAGUUUUCCCAGGAAUCCCU-3′, antisense: 5′-GGAUUCCUGGGAAAACUGGACUU-3′; mimic NC sense: 5′-UUCUCCGAACGUGUCACGUUU-3′, antisense: 5′-ACGUGACACGUUCGGAGAAUU-3′; miR-145-5p inhibitor: 5′-CUUAGCAUCUAAGGGAUUCCUGGG-3′; inhibitor NC: 5′-CGAACUUCACCUCGGCGCGGG-3′; si-ABRACL: 5′-GCGCUCACAGUAGGAGUUU-3′.

### 2.4. RNA Extraction and qRT-PCR

Total RNA was extracted from cells using TRIzol (Thermo Fisher Scientific, Waltham, MA, USA) and then reversely transcribed into complementary (cDNA) by cDNA synthesis kit (Thermo Fisher Scientific, Waltham, MA, USA).

Quantitative PCR was performed using miScript SYBR Green PCR Kit (Qiagen, Hilden, Germany) under following thermal cycling conditions: predenaturation at 95°C for 10 min, 40 cycles of 95°C for 15 s, and 60°C for 1 min [17]. Primers for miR-145-5p, U6, ABRACL, and GAPDH were purchased from GeneCopoeia (GeneCopoeia Inc., Guangzhou, China) and sequenced as below: miR-145-5p forward (stem-loop): 5′-TCGGCAGGGTCCAGTTTTCCCA-3′, reverse: 5′-CTCAAC TGGTGTCGTGGA-3′; U6 forward: 5′-GGAGCGAGATCCCTCCAAAAT-3′, reverse: 5′-GGCTGTTGTCATACTTCTCATGG-3′; ABRACL forward: 5′-ACCTCTTTGAAGCATTGGTAGG-3′, reverse: 5′-GCAGCTCTCCTGGATATGTTAC-3′; and GAPDH forward: 5′-GGAGCGAGATCCCTCCAAAAT-3′, reverse: 5′-GGCTGTTGTCATACTTCTCATGG-3′. The 2^−ΔΔCt^ value was used to compare the difference in relative expression of the target mRNA and miRNA between the control group and the experimental group. The experiment was repeated three times.

### 2.5. Western Blot

After transfection for 48 h, cells were washed 3 times with cold PBS. Whole cell lysate was added for cell lysate on ice for 10 min, and the BCA protein assay kit (Thermo, USA) was applied to determine protein concentration. A measure of 30 *μ*g of protein samples was processed for separation by polyacrylamide gel electrophoresis (PAGE) at a constant voltage of 80 V for 35 min followed by 120 V for 45 min, and sequentially transferred to polyvinylidene fluoride (PVDF) membranes (Amersham, USA). After blocked with 5% skim milk for 1 h at room temperature, the membranes were incubated with rabbit polyclonal anti-ABRACL antibody (Abcam, Cambridge, UK) and rabbit polyclonal anti-GAPDH antibody (Abcam, Cambridge, UK) overnight at 4°C. The membranes were washed 3 times with PBST (phosphate-buffered saline containing 0.1% Tween-20) for 10 min each time. Subsequently, the membranes were incubated with horseradish peroxidase- (HRP-) labeled secondary antibody goat anti-rabbit IgG H&L (Abcam, Cambridge, UK) for 1 h at room temperature. The membranes were washed 3 times with PBST buffer again. An optical luminometer (GE, USA) was employed to visualize protein bands, and the Image Pro Plus 6.0 (Media Cybernetics, USA) software was applied for further analysis.

### 2.6. CCK-8 Assay

Proliferative capability of EC cells was evaluated by CCK-8 assay. In short, Eca-109 cells (2 × 10^4^ cells/ml) were firstly inoculated into a 96-well plate for culture under the regular culture environment (5% CO_2_ and 37°C). Following 0, 24, 48, and 72 h, respectively, 10 *μ*l of CCK-8 reagent was added into each well for 2 h. After incubation, the optical density (OD) value at 450 nm of each well was measured using a microplate reader (Model 550; Bio-Rad Laboratories, Inc., Hercules, CA, USA). CCK-8 reagent used here was provided by the Cell Counting Kit-8 produced by Dojindo Molecular Technologies, Inc. (Kumamoto, Japan). The experiment was repeated three times.

### 2.7. Transwell Assay

#### 2.7.1. Transwell Migration Assay

Cancer cells in logarithmic growth phase were starved for 24 h at first. On the following day, cells were digested, centrifuged, and resuspended to a final concentration of 2 × 10^5^ cells/ml. A measure of 0.2 ml of cell suspension was added into Transwell upper compartment, while 700 *μ*l of precooled RPMI-1640 medium containing 10% FBS was added into the lower compartment. Cells were cultured in an incubator containing 5% CO_2_ at a temperature of 37°C. After 24 h, the cells still in the upper compartment were wiped off with a wet cotton swab, and the cells in the lower compartment were fixed using methanol for 30 min and then stained by 0.1% crystal violet for 20 min. Finally, cells were observed under an inverted microscope and five fields of view were randomly selected for cell count.

#### 2.7.2. Transwell Invasion Assay

A 24-well Transwell chamber (8 *μ*m in aperture, BD Biosciences) was used for cell invasion assay. Approximately 2 × 10^4^ cells were plated in the upper chamber which was precoated with Matrigel matrix (Corning, Corning, NY). RPMI-1640 medium containing 10% FBS was filled into the lower chamber. The following procedures were as similar as the above migration assay.

### 2.8. Dual-Luciferase Reporter Gene Assay

Eca-109 cells were seeded at 6 × 10^5^ cells/well in a 24-well plate and incubated for 24 h. The mutant (MUT) or wild-type (WT) 3′-UTR of ABRACL mRNA was amplified and then cloned into pmirGLO (Promega, WI, USA) to construct luciferase reporter vectors ABRACL-WT and ABRACL-MUT. Subsequently, ABRACL-WT and ABRACL-MUT were transfected into cells with miR-145-5p mimic or mimic NC by using Lipofectamine 2000, respectively. The Renilla luciferase vector pRL-TK (TaKaRa, Dalian, China) was used as a control reporter for normalization. After transfection, cells were cultured in RPMI-1640 medium containing 10% FBS. After 48 h, luciferase activities were measured using Dual-Luciferase Reporter Assay System Kit (Promega Corp., Madison, WI, USA). The experiment was repeated three times.

### 2.9. Statistical Analysis

All data were processed using SPSS 22.0 statistical software, and the measurement data were expressed in the form of the mean ± standard deviation. One-way analysis of variance (ANOVA) was used for pairwise comparison in data of multiple groups, while the *t*-test was implemented for data comparison between two groups. Student's *t*-test and one-way analysis of variance (ANOVA) were applied for analyzing the comparisons between two groups and among multiple groups. *p* < 0.05 indicates statistically significant difference.

## 3. Results

### 3.1. miR-145-5p Is Poorly Expressed in EC Cells

**Figure 1:**
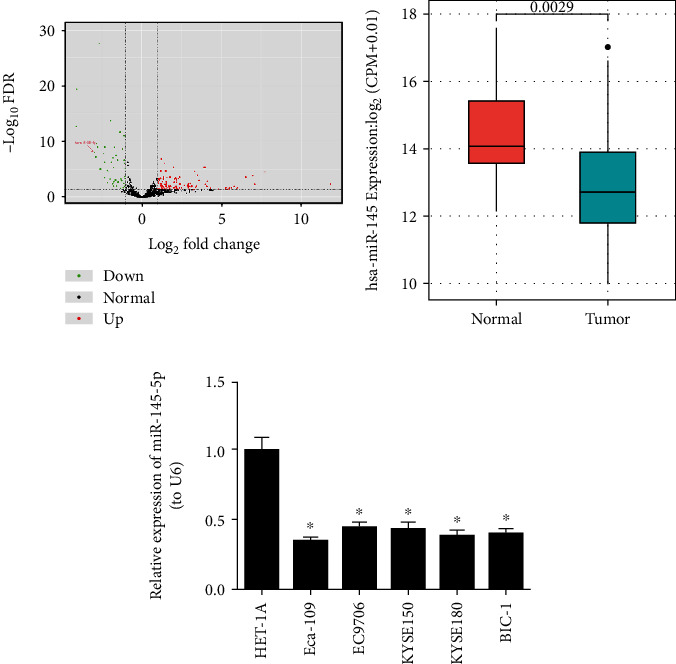
miR-145-5p is poorly expressed in EC tissue and cells. (a) Volcano plot shows the DEmiRNAs in TCGA-ESCA dataset. (b) Relative expression of miR-145-5p in EC samples in TCGA-ESCA dataset. (c) qRT-PCR shows the relative expression of miR-145-5p in EC cell lines Eca-109, EC9706, KYSE150, KYSE180, and BIC-1 and in normal esophageal cell line HET-1A. ∗ indicates *p* < 0.05.

A total of 158 differentially expressed miRNAs (DEmiRNAs) were screened via differential analysis using the “edgeR” package (Figure 1(a)). Among the DEmiRNAs, miR-145-5p was found to be significantly decreased in EC tumor tissues (*n* = 153) relative to that in normal tissues (*n* = 11) in TCGA database (Figure 1(b)). In addition, EC cell lines Eca-109, EC9706, KYSE150, KYSE180, and BIC-1 and normal esophageal cell line HET-1A were collected to examine miR-145-5p expression. qRT-PCR was performed and it was showed that compared with HET-1A, EC cells especially Eca-109 cells had significantly reduced expression of miR-145-5p (Figure 1(c)). Thus, Eca-109 cell line was chosen for subsequent experiments for further analysis.

### 3.2. miR-145-5p Inhibits the Proliferation, Migration, and Invasion of EC Cells

**Figure 2:**
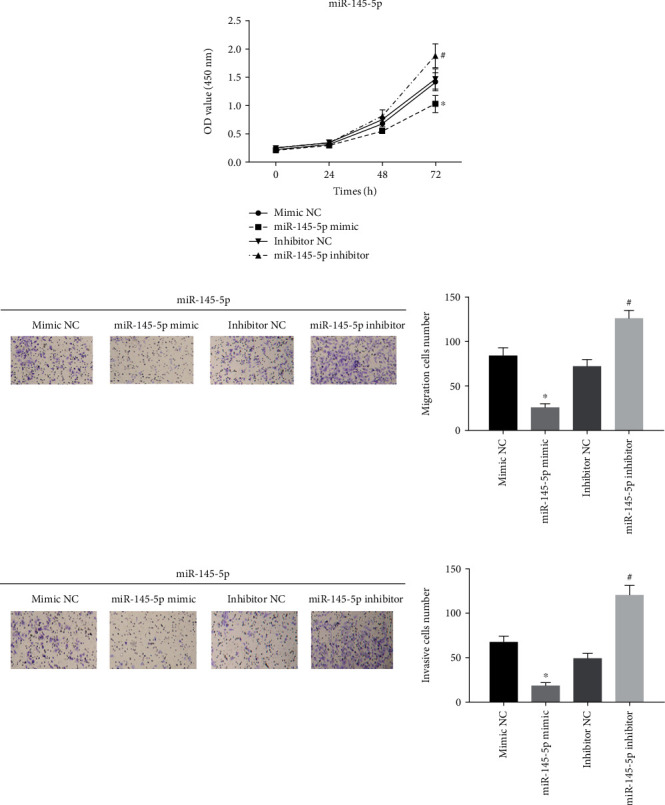
miR-145-5p inhibits the proliferation, migration, and invasion of EC cells. miR-145-5p mimic, miR-145-5p inhibitor, mimic NC, and inhibitor NC were transfected into Eca-109 cells. (a) CCK-8 assay and (b) migration and (c) invasion assays (Transwell (100x)) show cell proliferative, migratory, and invasive abilities in each treatment group. ∗ indicates *p* < 0.05 relative to mimic NC; # indicates *p* < 0.05 relative to inhibitor NC.

A series of functional experiments were conducted to identify the mechanism by which miR-145-5p regulates EC cell biological behaviors. Mimic NC, miR-145-5p mimic, inhibitor NC, and miR-145-5p inhibitor were transfected into Eca-109 cells. CCK-8 assay suggested that the proliferative ability of Eca-109 cells was greatly decreased in the miR-145-5p mimic group relative to that in the NC group, but significantly increased in the miR-145-5p inhibitor group (Figure 2(a)). Similarly, cell migratory and invasive abilities were both reduced in the miR-145-5p mimic group but enhanced in the miR-145-5p inhibitor group relative to those in the NC groups, as judged by Transwell migration and invasion assays (Figures 2(b) and 2(c)). Overall, all results indicated that high expression of miR-145-5p could inhibit the proliferation, migration, and invasion of EC cells.

### 3.3. ABRACL Is a Direct Target of miR-145-5p

**Figure 3:**
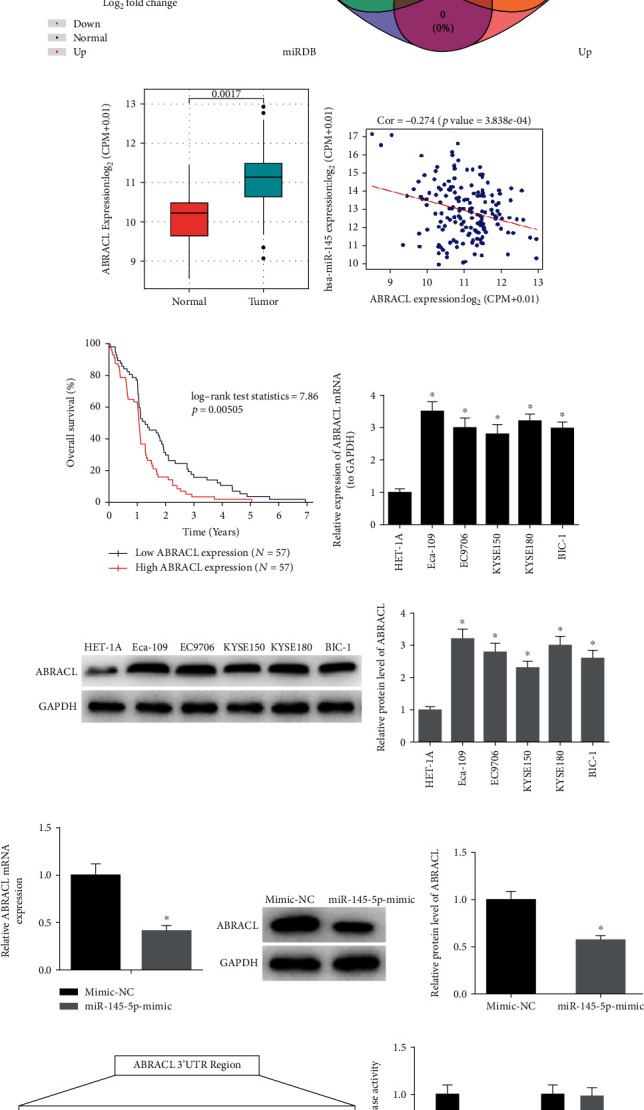
ABRACL is a direct target of miR-145-5p. (a) Volcano plot shows the DEmRNAs in TCGA-ESCA dataset. (b) Venn diagram shows the overlapping mRNAs between the predicted targets of miR-145-5p and the upregulated DEmRNAs in TCGA-ESCA dataset. (c) Relative expression of ABRACL in the samples obtained from TCGA-ESCA dataset. (d) The correlation between miR-145-5p and ABRACL. (e) The survival analysis for ABRACL in TCGA-ESCA dataset (each vertical node represents a death of a follow-up individual), (f, g) qRT-PCR and Western blot show the relative mRNA and protein levels of ABRACL in EC cell lines Eca-109, EC9706, KYSE150, KYSE180, and BIC-1 and in normal esophageal cell line HET-1A. (h, i) The relative mRNA and protein expression of ABRACL in mimic NC and miR-145-5p mimic groups. (j) Putative binding sites of miR-145-5p on ABRACL 3′-UTR along with corresponding mutation sites and relative luciferase activity in each treatment group. ∗ indicates *p* < 0.05.

Differential analysis showed that there were 3,224 differentially expressed mRNAs (DEmRNAs) identified in TCGA-ESCA dataset (Figure 3(a)). TargetScan, miRDB, and miRTarBase databases were applied to predict target mRNAs of miR-145-5p, and the results were then mapped into a Venn diagram with the upregulated DEmRNAs in TCGA-ESCA dataset. Eventually, 10 overlapping mRNAs were obtained (Figure 3(b)). Among the 10 mRNAs, four genes including TGFBR2, ABRACL, ZBTB47, and MASTL were detected to be significantly associated with the prognosis of EC patients as revealed by survival analysis based on the clinical samples (*n* = 204) obtained from TCGA-ESCA dataset. Meanwhile, ABRACL was shown to be the most elevated in EC tissue samples (*n* = 176) relative to that in normal tissue samples (*n* = 13) (Figure 3(c)). Additionally, Pearson correlation analysis suggested that there was a negative correlation between the expression levels of miR-145-5p and ABRACL (Figure 3(d)). In order to validate whether ABRACL is associated with the survival of patients with EC, a total of 114 samples (including 57 samples with low ABRACL and 57 samples with high ABRACL) and corresponding complete clinical data were accessed from TCGA database and then used for survival analysis (Figure 3(e)). Results showed that high expression of ABRACL was able to predict poor prognosis. In the meantime, the expression of ABRACL was test in EC cell lines. Compared with HET-1A cell line, EC cell lines had remarkably elevated expression of ABRACL (Figures 3(f) and 3(g)).

To further understand the regulatory effect of miR-145-5p on ABRACL, the expression of ABRACL was detected in cells with miR-145-5p overexpression. The results showed that overexpression of miR-145-5p significantly decreased the expression of ABRACL (Figures 3(h) and 3(i)). Additionally, TargetScan database was consulted and it was shown that there were potential binding sites of miR-145-5p on ABRACL 3′-UTR (Figure 3(j)). Dual-luciferase reporter assay was conducted for further verification and indicated that overexpression of miR-145-5p significantly decreased the luciferase activity of Eca-109 cells cotransfected with ABRACL-WT but had no effect in cells containing ABRACL-MUT. Collectively, these results supported the notion that ABRACL was a direct target of miR-145-5p.

### 3.4. miR-145-5p Regulates ABRACL to Affect the Proliferation, Migration, and Invasion of EC Cells

**Figure 4:**
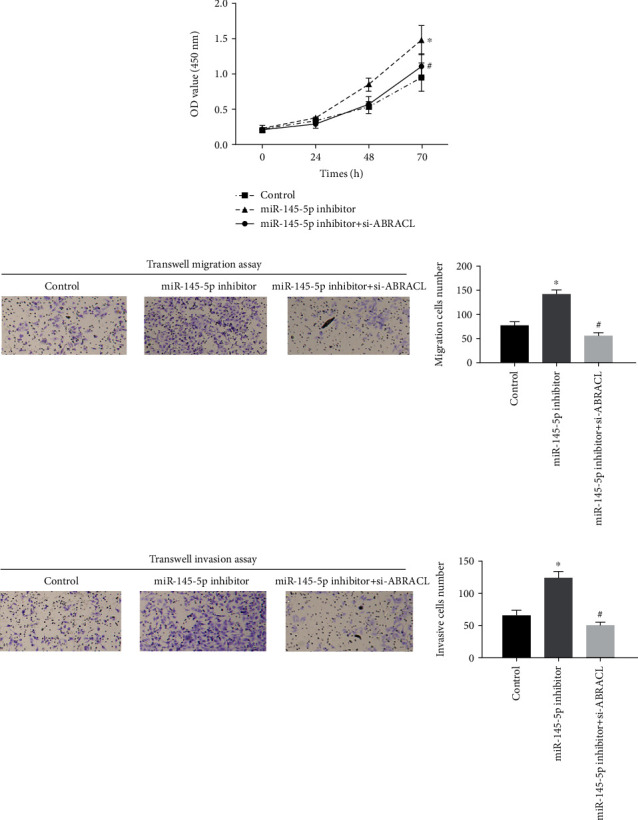
miR-145-5p functions on the proliferation, migration, and invasion of EC cells via targeting the expression of ABRACL. Control, miR-145-5p inhibitor, and miR-145-5p inhibitor+si-ABRACL were transfected into Eca-109 cells. (a) CCK-8 assay and (b) migration and (c) invasion assays (Transwell (100x)) show cell proliferative, migratory, and invasive abilities in each treatment group. ∗ indicates *p* < 0.05 relative to the control group; # indicates *p* < 0.05 relative to the miR-145-5p inhibitor group.

To further explore the regulatory mechanism by which miR-145-5p targets ABRACL to mediate cell biological behaviors of EC, rescue experiments were performed on Eca-109 cells. Since we had verified that low expression of miR-145-5p could promote cell proliferation, we then simultaneously transfected si-ABRACL and miR-145-5p inhibitor into Eca-109 cells. The results revealed that the cell proliferative ability was significantly decreased after cotransfection compared to that of the miR-145-5p inhibitor group (Figure 4(a)). Similarly, cell migration and invasion were noted to show similar changes, as detected by Transwell migration and invasion assays (Figures 4(b) and 4(c)). It was suggested that miR-145-5p affected the physiological activities of cancer cells by regulating the expression of ABRACL.

## 4. Discussion

miRNAs compose a large family widely present in eukaryotic cells and some of them harbor a targeting relationship with mRNAs. Genomic analysis showed that there are over 5,300 human genes that targeted by miRNAs, accounting for 30% of all human genes, and their expression shows an intimate correlation with cancers [18, 19]. Studies revealed that there are many miRNAs altered between EC patients and healthy people. miR-145 was reported to be significantly downregulated in ESCC tissue and cell lines and play an important role in inhibiting cancer cell proliferation, migration, and metastasis. For example, miR-145 can directly target the 3′-UTR of PLCE1, and the downregulation of PLCE1 induces cell apoptosis as well as enhances the sensitivity of tumor cells to chemotherapeutic drugs [20]. Meanwhile, miR-145 is able to inhibit the growth of ESCC cells by targeting c-Myc [21]. In addition, there is a study showing that miR-145 can target and interact with connective tissue growth factor (CTGF) to affect EC cell proliferation, migration, invasion, and epithelial-mesenchymal transition (EMT) process [22]. In our study, we also observed that the proliferation, migration, and invasion of EC cells were affected significantly after miR-145-5p was overexpressed or inhibited, and high miR-145-5p negatively worked in EC progression. To further analyze the mechanism by which miR-145-5p regulates cell activities of EC, target prediction was performed and it was found that ABRACL was a potential target of miR-145-5p. Additionally, survival analysis revealed that there was a close relationship between ABRACL and the prognosis of EC patients, and dual-luciferase reporter gene assay further validated that miR-145-5p could target and bind with ABRACL to suppress its expression. Therefore, we speculated that miR-145-5p mediated EC progression probably via targeting the expression of ABRACL.

ABRACL, a winged-helix protein, plays an important role in a variety of developmental processes. A comparative expression analysis of ABRACL with Dlx2, cyclinD2, and Lhx6 revealed that ABRACL is restricted in the proliferating cell population in the subventricular zone within ganglionic eminences, with a pattern similar to that of cyclinD2 [14]. In addition, ABRACL was detected to be upregulated in gastric cancer tissue compared to that in normal gastric tissue, which is associated with poor prognosis. Meanwhile, an enrichment analysis revealed that ABRACL is mostly enriched in some signaling pathways, such as cell cycle, TNFR pathway, proteasome degradation, and mitochondrial pathway [16]. Besides, studies found that ABRACL bears a similar structure to ABD2 and may be a transcriptional synergistic activator [13, 23, 24] closely related to cell cycle [25]. In this study, dual-luciferase reporter assay demonstrated that ABRACL was a direct target of and negatively regulated by miR-145-5p. Rescue experiments suggested that the effect of silenced miR-145-5p on cell proliferation, migration, and invasion could be attenuated upon ABRACL knockdown. These results suggested that miR-145-5p regulated the cellular functions of EC cells by targeting ABRACL.

In conclusion, our study first proposes that miR-145-5p targets ABRACL to inhibit the proliferation, migration, and invasion of EC cells, which helps to provide a novel therapeutic target for future EC treatment.

## Data Availability

The data and materials in the current study are available from the corresponding author on reasonable request.

## References

[1] Maghsudlu M., Farashahi Yazd E. (2017). Heat-induced inflammation and its role in esophageal cancer. *Journal of Digestive Diseases*.

[2] Chen W., Zheng R., Baade P. D. (2016). Cancer statistics in China, 2015. *CA: a Cancer Journal for Clinicians*.

[3] Bohanes P., Yang D., Chhibar R. S. (2012). Influence of sex on the survival of patients with esophageal cancer. *Journal of Clinical Oncology*.

[4] Kamangar F., Dores G. M., Anderson W. F. (2006). Patterns of cancer incidence, mortality, and prevalence across five continents: defining priorities to reduce cancer disparities in different geographic regions of the world. *Journal of Clinical Oncology*.

[5] Baer C., Squadrito M. L., Laoui D. (2016). Suppression of microRNA activity amplifies IFN-*γ*-induced macrophage activation and promotes anti-tumour immunity. *Nature Cell Biology*.

[6] Croce C. M. (2009). Causes and consequences of microRNA dysregulation in cancer. *Nature Reviews. Genetics*.

[7] Zeng B., Zhang X., Zhao J. (2019). The role of DNMT1/hsa-miR-124-3p/BCAT1 pathway in regulating growth and invasion of esophageal squamous cell carcinoma. *BMC Cancer*.

[8] Fan Y. X., Bian X. H., Qian P. D. (2018). MicroRNA-125b inhibits cell proliferation and induces cell apoptosis in esophageal squamous cell carcinoma by targeting BMF. *Oncology Reports*.

[9] Chen Q., Zhou L., Ye X., Tao M., Wu J. (2020). miR-145-5p suppresses proliferation, metastasis and EMT of colorectal cancer by targeting CDCA3. *Pathology - Research and Practice*.

[10] Tang W., Zhang X., Tan W. (2019). miR-145-5p suppresses breast cancer progression by inhibiting SOX2. *The Journal of Surgical Research*.

[11] Zhou T., Chen S., Mao X. (2019). miR-145-5p affects the differentiation of gastric cancer by targeting KLF5 directly. *Journal of Cellular Physiology*.

[12] Pang T. L., Chen F. C., Weng Y. L. (2010). Costars, a Dictyostelium protein similar to the C-terminal domain of STARS, regulates the actin cytoskeleton and motility. *Journal of Cell Science*.

[13] Lin J., Zhou T., Wang J. (2011). Solution structure of the human HSPC280 protein. *Protein Science*.

[14] Stylianopoulou E., Kalamakis G., Pitsiani M. (2016). HSPC280, a winged helix protein expressed in the subventricular zone of the developing ganglionic eminences, inhibits neuronal differentiation. *Histochemistry and Cell Biology*.

[15] Ura B., Monasta L., Arrigoni G. (2017). A proteomic approach for the identification of biomarkers in endometrial cancer uterine aspirate. *Oncotarget*.

[16] Wang D., Liu H. Q., Ren C., Wang L. (2019). High expression of ABRACL is associated with tumorigenesis and affects clinical outcome in gastric cancer. *Genetic Testing and Molecular Biomarkers*.

[17] Mei L. L., Wang W. J., Qiu Y. T., Xie X. F., Bai J., Shi Z. Z. (2017). miR-145-5p suppresses tumor cell migration, invasion and epithelial to mesenchymal transition by regulating the Sp1/NF-*κ*B signaling pathway in esophageal squamous cell carcinoma. *International Journal of Molecular Sciences*.

[18] Calin G. A., Sevignani C., Dumitru C. D. (2004). Human microRNA genes are frequently located at fragile sites and genomic regions involved in cancers. *Proceedings of the National Academy of Sciences of the United States of America*.

[19] Lewis B. P., Burge C. B., Bartel D. P. (2005). Conserved seed pairing, often flanked by adenosines, indicates that thousands of human genes are microRNA targets. *Cell*.

[20] Cui X. B., Li S., Li T. T. (2016). Targeting oncogenic PLCE1 by miR-145 impairs tumor proliferation and metastasis of esophageal squamous cell carcinoma. *Oncotarget*.

[21] Wang F., Xia J., Wang N., Zong H. (2013). miR-145 inhibits proliferation and invasion of esophageal squamous cell carcinoma in part by targeting c-Myc. *Onkologie*.

[22] Han Q., Zhang H. Y., Zhong B. L., Wang X. J., Zhang B., Chen H. (2016). MicroRNA-145 inhibits cell migration and invasion and regulates epithelial-mesenchymal transition (EMT) by targeting connective tissue growth factor (CTGF) in esophageal squamous cell carcinoma. *Medical Science Monitor*.

[23] Fogl C., Puckey L., Hinssen U. (2012). A structural and functional dissection of the cardiac stress response factor MS1. *Proteins*.

[24] Galkin V. E., Orlova A., Cherepanova O., Lebart M. C., Egelman E. H. (2008). High-resolution cryo-EM structure of the F-actin-fimbrin/plastin ABD2 complex. *Proceedings of the National Academy of Sciences of the United States of America*.

[25] Baumann D. G., Dai M. S., Lu H., Gilmour D. S. (2018). GFZF, a GlutathioneS-Transferase protein implicated in cell cycle regulation and hybrid Inviability, is a transcriptional coactivator. *Molecular and Cellular Biology*.

